# SppR in *Streptococcus mutans* coordinates stress tolerance response and BrpA expression

**DOI:** 10.3389/fcimb.2026.1907905

**Published:** 2026-07-27

**Authors:** Zezhang T. Wen, Bo Yang, Lauren C. Guillot, Lin Zeng

**Affiliations:** 1 Department of Oral and Craniofacial Biology, Louisiana State University Health School of Dentistry, New Orleans, LA, United States; 2 Department of Microbiology, Immunology and Parasitology, Louisiana State University Health School of Medicine, New Orleans, LA, United States; 3 Department of Oral Biology, College of Dentistry, University of Florida, Gainesville, FL, United States

**Keywords:** biofilm formation, brpA expression, oxidative stress tolerance response, S. mutans, SppR

## Abstract

SppR is a transcriptional repressor of fructanase FruA and fructose-1-phosphatase SppA. In this study, a frameshift *sppR* mutant was constructed and analyzed using *in vitro* biofilm models and stress challenge assays for its role in *S. mutans’* stress tolerance response and biofilm formation. In addition, luciferase reporter fusion assay and *in vitro* transcription assay were used to analyze the role of SppR in regulation of *brpA*. Results showed that *sppR* deficiency caused no major effect on planktonic growth and cell morphology in regular BHI broth, however negatively impacted growth at low pH and in the presence of oxidative stressor (*P* < 0.001). Relative to the parent strain, UA159, the *sppR* mutant also had a reduced survival rate following acid killing (P<0.01) and hydrogen peroxide challenge (P<0.001). Luciferase reporter assays showed that *sppR* deficiency reduced *brpA* promoter-reporter activity by >2-fold (*P<*0.05), while the disruption of a putative SppR-binding site in the promoter reduced its activity by >7-fold (P<0.001). A recombinant SppR (rSppR) retarded the migration of a *brpA* promoter-containing probe during electrophoresis and enhanced the transcription of *brpA in vitro.* RNA-seq analysis also showed that deficiency of SppR led to altered expression of >136 genes by >2-fold (*P<*0.05), including 68 genes up-regulated and 68 genes reduced. Among the down-regulated genes were those belonging to DNA-repair and acid tolerance response. These results suggest that SppR plays an important role in *S. mutans* physiology including acid and oxidative stress tolerance response and regulation of BrpA expression.

## Introduction

*S. mutans* is a keystone pathogen of human dental caries that is known for its abilities to colonize and persist on the tooth surface, utilize various sugars at micro-concentrations producing organic acids, survive and adapt to harsh environmental stressors, such as low pH, and become significant under certain conditions leading to the development of carious lesions. It possesses multiple pathways to uptake sugars, including the sugar-specific phosphoenolpyruvate:sugar phosphotransferase system (PTS) and the non-sugar-specific multiple sugar metabolism (*msm*) ABC transport system (Russell et al., 1992; Vadeboncoeur and Pelletier, 1997). There are at least 14 sugar-specific PTS enzyme II permeases that are capable of transporting a variety of mono- and di-saccharides, including glucose, sucrose, mannose, fructose, cellobiose, lactose, galactose, N-acetyl-glucosamine, glucosamine, and maltose (Ajdic et al., 2002; Lemos et al., 2019). The MSM system is also capable of internalizing raffinose, melibiose, and isomaltosaccharides. Like *Bacillus* spp., sugar metabolism in *S. mutans* is highly regulated by substrate induction and carbon catabolite repression, which controls the ability of a bacterium to catabolize more-preferred sugars over non-preferred sugars in the environment (Abranches et al., 2008; Zeng et al., 2013); and carbon catabolite repression is controlled primarily by the HPr component of the PTS system and the LacI-family catabolite control proteins CcpA and others like GlcR (Stulke et al., 2001; Abranches et al., 2008). Unlike *Bacillus* spp., however, carbon catabolite repression in *S. mutans* is dominantly controlled by the glucose/mannose EII permease, in particular ManL (EIIAB^Man^), in concert with HPr (Zeng et al., 2006; Zeng and Burne, 2013). As such, transcriptomic analysis of target mutants, along with function assays and animal model studies have consistently shown that sugar sources and availability can dramatically influence the metabolic pathways and consequently cariogenicity of *S. mutans*.

*S. mutans* lives primarily in the high-density, high-diversity microbiota (aka dental plaque) on the tooth surface, exposed to harsh and fluctuating conditions including pH, nutrient availability, and various antimicrobial agents like SDS, chlorhexidine, toxic metals, hydrogen peroxide and bacteriocins. *S. mutans* deploys a layered network of molecular defenses that coordinate protein quality control, environmental sensing, and global transcriptional regulations.

Biofilm regulatory protein BrpA is a surface-associated multifunctional protein that plays a central role in stress tolerance response, particularly against cell envelope stressors, and in biofilm development, most notably under sucrose-rich growth conditions (Wen et al., 2006; Bitoun et al., 2012b; Wen et al., 2016; Wen et al., 2018). Loss of BrpA as a result of allelic exchange mutagenesis profoundly compromises the pathogenic potential of the deficient mutant: in a rat caries model, *S. mutans* lacking BrpA shows an almost complete loss of cariogenicity compared with the wild-type strain (Wen et al., 2018). Beyond its regulatory functions, BrpA belongs to the conserved LytR-CpsA-Psr (LCP) family of proteins, which are phosphotransferases/ligases in low G+C Gram-positive bacteria that catalyze the attachment of the cell wall-associated polymers like capsules to the peptidylglycan. In *S. mutans*, BrpA, together with its paralogue Psr (to a lesser extent), catalyze the attachment of rhamnose-glucose polymers (RGP) to the peptidoglycan scaffold, a process that profoundly influences cell surface architecture, envelope integrity, and overall bacterial physiology in *S. mutans* (De et al., 2017; Wen et al., 2018). Recent studies have shown that multiple *cis*- and *trans*-acting elements are involved in regulation of *brpA* expression, including a Fnr-box as a major repressor (**Supplementary Figure 1**) (Liao et al., 2017).

SppR (formerly GlcR) is a member of the DeoR-family transcriptional regulators, which are well documented for their roles in regulation of genes like those for glucosidase in *B. subtilis* (Stulke et al., 2001) and fructanase FruA of *S. mutans* (Wen and Burne, unpublished data) via carbon catabolite repression. In *B. subtilis, glcR* is co-transcribed with *phoC* (formerly *ywpJ*) that encodes an acid phosphatase (Morabbi Heravi et al., 2019), which can dephosphorylate mannose-6-phosphate. Accumulation of mannose-6-phosphate also led to de-repression of the *glcR-phoC* operon. In *S. mutans.* SppR regulates phosphosugar stress response with the intercellular level of fructose-1-phosphate (F-1-P) serving as the signal (Zeng and Burne, 2019). The gene *sppR* is co-transcribed as a bicitronic operon with downstream *sppA*, which encodes a hexose-phosphate phosphohydrolase. Accumulation of F-1-P, as a result of fructose metabolism, not only increased *sppA* expression, but also caused significant alterations in biofilm formation as a result of cell lysis and extracellular DNA secretion. In this study, luciferase reporter fusion assay, along with transcriptomics (RNA-seq) and function assays were used to further characterize the role of SppR in *S. mutans* physiology mediated through *brpA* expression. The results showed SppR in *S. mutans* is a positive regulator of *brpA* expression that modulates acid and oxidative stress tolerance in response to phosphosugar signals.

## Materials and methods

### Bacterial strains and cultivation

All bacterial strains created and used in this study were listed in **Table 1**. Unless it is specified otherwise, *S. mutans* UA159 and its derivatives including target mutants and reporter fusion strains were maintained and grown in Brain Heart Infusion (BHI; Becton, Dickinson and Company, Sparks, MD, USA) in a 37 °C aerobic chamber plus 5% CO_2_. For agar medium plates, Bacto agar (Becton, Dickinson and Company, Sparks, MD, USA) was added at 1.5% w/v. When it was necessary, the growth medium was supplemented with antibiotics erythromycin (Sigma, St Louis, MO, USA) (10 μg mL^−1^), spectinomycin (Sigma) (1 mg mL^−1^), and/or kanamycin (Sigma) (1 mg mL^−1^). *Escherichia coli* strains were grown in an aerobic incubator at 37°C in LB broth with shaking at 200 rpm and supplemented with antibiotics erythromycin (Sigma, St Louis, MO, USA) (300 μg mL^−1^), spectinomycin (Sigma) (100 μg mL^−1^), ampicillin (100 μg mL^-1^), and/or kanamycin (Sigma) (40 μg mL^−1^), if necessary.

**Table 1:** Bacterial strains and plasmids used in this study.

Strains/Plasmid	Major characteristics	References
*S. mutans* UA159	wild-type	ATCC700610
*S. mutans* ΔsppR	UA159/Δ*sppR*, Erm^r^	This study
*S. mutans* ΔsppR/c	UA159/Δ*sppR/gtfA::glcR*, Erm^r^, Kan^r^	This study
*S. mutans* SppRf	UA159/*sppR*ΔC62	This study
*S. mutans* SppRf/c	UA159/*sppR*ΔC62/pIB::*sppR*, Erm^r^	This study
*E. coli* DH10B	Cloning host, *mcrA, mcrBC, mrr*, and *hsd*	Invitrogen, Inc.
*E. coli* M15	Expression host, Kan^r^	Qiagen, Inc.
pQE30	Expression vector, Amp^r^	Qiagen, Inc.
pFW11-*luc*	Integration vector containing a promoterless luciferase gene, Spc^r^	(Kreth et al., 2005)
pBGK3	Integration vector	(Liao et al., 2017)
pFW11::Pldh::luc	Luciferase reporter fused with the lactate dehydrogenase gene promoter *Pldh*, Spc^r^	(Bitoun et al., 2012a)
SP3	pFW11::P*brpA::luc*, SppR-box1: AaGatGGAGTAtTTcTTGATgtTTA, box2: AaGtgATAGAgCTTgTGATGAtTTt	This study
sb1d1	SP3 with GATGTTTACCTT of the SppR-box replaced by ctcgag	This study
sb1d2	SP3 with GGAGTTTGATgtTTA of the SppR-box1 deleted	This study
sb1m	SP3 with SppR-box1 mutations to taatgATAGAgaaagTGATGttaat	This study
sb2d	SP3 with SppR-box2 deletion of AaGtgATAGATGATG	This study
sb2m	Sp3 with SppR-box2 mutations of ACgCTGTT to taGCTaaa	This study
pIB184	Shuttle expression vector for *S. mutans*, Erm^r^	(Biswas et al., 2008)
pIB184:*sppR*	pIB184::*sppR*	This study

Kan^r^, Erm^r^ and Spc^r^ for kanamycin, erythromycin and spectinomycin resistance, respectively.

### Construction and characterization of an unmarked *sppR* mutant

Allelic exchange *sppR* mutant was constructed using the PCR-Ligation-mutation strategy with part of the coding sequence replaced by an erythromycin-resistance marker (see **Supplementary Table 1** for primers used) and further verified by Sanger sequencing (Wen and Burne, 2002). Considering *sppR* is co-transcribed with down-stream *sppA* as bicistronic operon, a markerless *sppR* mutant was also constructed by deleting the cytosine at nucleotide 62 of the coding sequence, resulting in a frame-shift mutation of *sppR* by following strategies as described previously (Junges et al., 2023; Ellepola et al., 2024b). For the *sppR* mutant complementation, the wild-type *sppR* gene plus its cognate promoter was PCR amplified and integrated via double crossover recommendations at the *gtfA* site via integration vector pBGK (De et al., 2017; Zeng and Burne, 2019). All related molecular bioagents were purchased from New England Biolabs (Beverly, MA, USA) and/or Invitrogen (Thermo Fisher Scientific, Life Technologies, NY, USA) and used as recommended by the suppliers.

Growth characteristics of the *sppR* deficient mutants were analyzed using a Bioscreen C with bacterial strains grown in regular BHI broth, BHI adjusted to pH 6.0, BHI with inclusion of methyl viologen at 25 mM and 12.5 mM, and BHI with sodium nitroprusside at 2 mM (Ellepola et al., 2021). For the impact of *sppR* on *S. mutans* stress tolerance response, acid killing by incubation in 0.1 M glycine buffer at pH 2.8 and hydrogen peroxide challenge assay with inclusion of hydrogen peroxide at 58 mM were carried out as previously described (Wen et al., 2005). For biofilm formation, *S. mutans* strains were grown in biofilm medium with glucose (20 mM), sucrose (20 mM), and glucose plus sucrose (18, 2 mM), and bacterial biofilms were allowed to grow on polystyrene surface in 96-well plates and measured using a spectrophotometer following 0.1% crystal violet staining or on hydroxyapatite (HA) discs vertically deposited in 96 well plates and analyzed using a confocal laser scanning microscope as described previously (Wen and Burne, 2002; Bitoun et al., 2013).

### Luciferase reporter fusion assay

To assess the role of *sppR* in *brpA* expression, the *sppR* mutant was transformed with luciferase reporter fusion constructs of the intact *brpA* promoter and its derivatives with putative GlcR-boxes’ deletion or site-directed mutations (**Supplementary Figure 1**) (Liao et al., 2017), and transformants carrying the reporter fusions were isolated from BHI agar plates supplemented with spectinomycin. The impact of *sppR*-deficiency on *brpA* expression was analyzed by luciferase assays with the wild-type strains as the control (Liao et al., 2017). *S. mutans* strains carrying the luciferase reporter fused with the constitutive promoter of the lactate dehydrogenase gene *ldh* was also used as a reference (**Table 1**).

### Recombinant protein expression and purification

For expression and purification of recombinant SppR protein (rSppR), *sppR* (SMU.507) was amplified by PCR using high fidelity DNA polymerase Q5 (NEB Biolabs) (**Supplementary Table 1**) and cloned in pQE30 (Qiagen, Inc.). Following Sanger sequencing to confirm the accuracy of the cloned DNA, rSppR was expressed in *E. coli* M15 by isopropyl β-D-1-thiogalactopyranoside induction and the N-terminal His-tagged fusion protein was then purified using nickel resins (Fisher Scientific) under native conditions similarly as described previously (Bitoun et al., 2012b). The purified rSppR was re-constituted in phosphate buffered saline, pH 7.6 plus 20% glycerol using Amicon-10 Centrifugal Filters (10 kDa MWCO, Millipore).

### Electrophoretic mobility shift assay and *in vitro* transcription assay

Electrophoretic mobility shift assay (EMSA) was used to examine if SppR interacts with a fragment containing the *brpA* promoter (**Supplementary Table 1**); and DNA mobility shift as a result of DNA-protein interaction was visualized via LightShift Chemiluminescent EMSA Kit (Pierce, Rockford, IL) (Bitoun et al., 2012b; Ellepola et al., 2024a). For the role of SppR in *brpA* expression, *in vitro* transcription assay (IVT) was also done using native *S. mutans* RNA polymerase and recombinant sigma factor A (SigA) with and without inclusion of rSppR as described previously (Ellepola et al., 2024a). The resulting transcripts were separated using 1.2% denaturing agarose gel, stained using SYBR Gold (InVitrogen, CA) and imaged using a GelDoc GO system (BioRad).

### Transcriptomics analysis

Transcriptional profiling via RNA-sequencing was carried out as described previously (Zeng et al., 2013; Wen et al., 2017). For total RNAs, *S. mutans* wild-type and its *sppR* mutant were growing in regular BHI broth until mid-exponential phase with OD_600nm_ ~0.4 and immediately treated with RNAProtect (Qiagen Inc., CA) prior to RNA extraction using a ZymoBIOMICS Quick-bacteria and fungi RNA Miniprep Kit (Zymo Research) (Wen et al., 2006; Wen et al., 2017). Following ribosomal RNA depletion, libraries were generated using Illumina’s Stranded Total RNA Prep Ligation with Ribo-Zero Plus kit and then sequenced at the SeqCenter (Pittsburgh, PA) using an Illumina NovaSeq X Plus platform, producing paired end 150 bp reads. Then, the adapters were removed, reads were processed, cleaned and mapped the *S. mutans* UA159 genome (GCF_000007465.2/) with HISAT2 (v2.2.1) using default parameters + “very sensitive” (Kim et al., 2019). Differentially expressed genes were identified using edgeR’s glmQLFTest with those with a |log_2_(fold change)| > 1 and a *P* < 0.05. Selected genes were further analyzed using RealTime-qPCR with 16S rRNA as the control (Wen et al., 2006; Wen et al., 2017). Gene expression data are deposited in the NCBI Gene Expression Omnibus (GEO) database (www.ncbi.nlm.nih.gov/geo) under GEO Series Accession number GSE328150.

### Statistical analysis

All experiments were done with independent repeats of at least three times with proper controls. The results were analyzed using Student *t*-test for differences between different groups/conditions unless specified otherwise; and a difference of *P* < 0.05 was considered statistically significant.

## Results

### SppR-deficiency affects oxidative stress and acid tolerance of *S. mutans*

**Figure 1:**
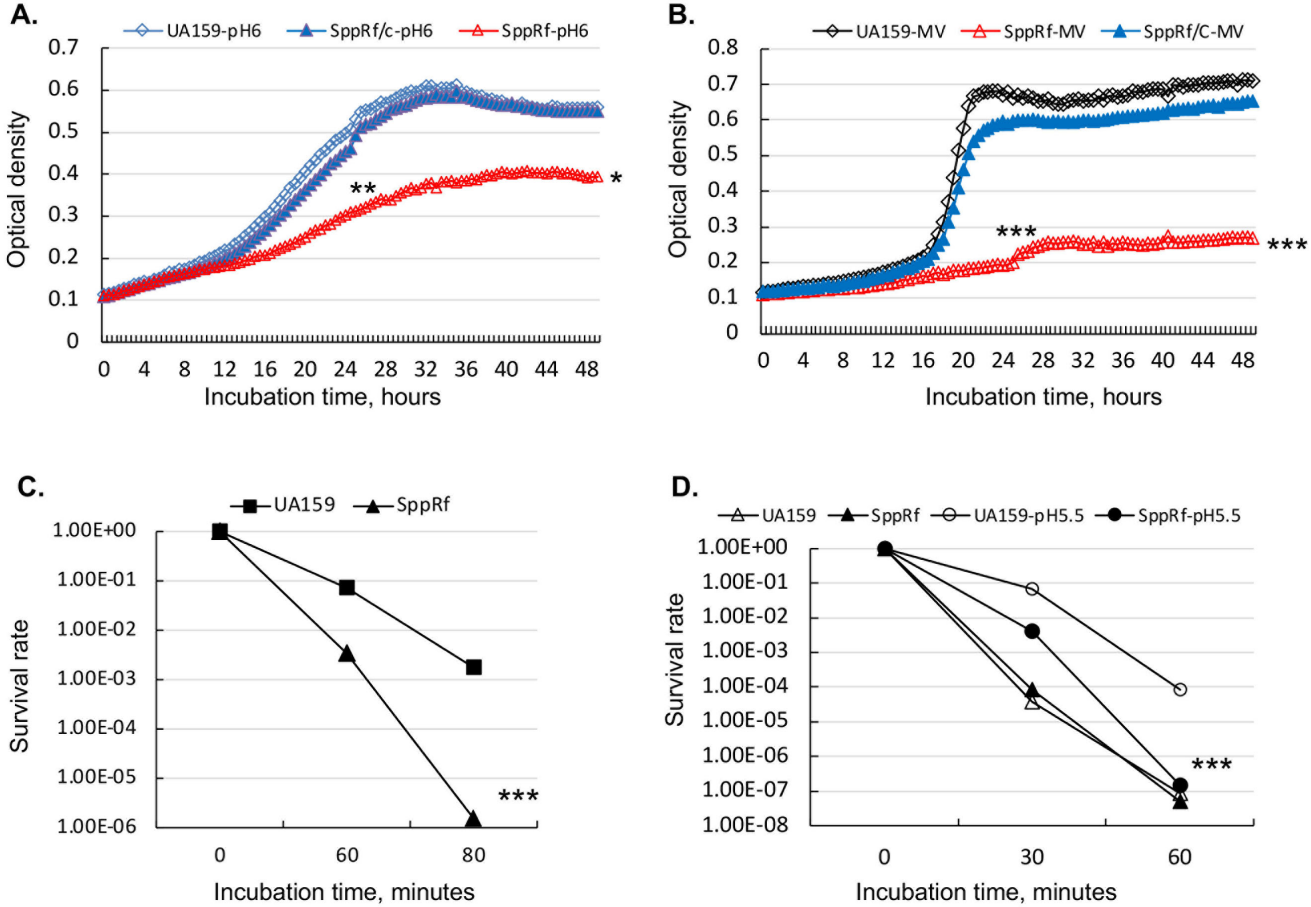
Growth characterization **(A, B)**, hydrogen peroxide challenge **(C)**, and acid killing assay **(D)** of the *sppR* mutant. For growth characteristics, *S. mutans* UA159 and its markerless *sppR* mutant (SppRf), and complemental strain (SppRf/c) were grown in BHI adjusted to pH 6.0 **(A)** and BHI with inclusion of methyl viologen (25 mM, MV) **(B)** in a Bioscreen C system. For oxidative stress tolerance, regular BHI-grown *S. mutans* strains were incubated in 0.1 M glycine buffer, pH 7.0 containing 58 mM hydrogen peroxide for periods of time as indicated **(C)**. For acid tolerance response, regular BHI-grown *S. mutans* UA159 and SppRf were incubated in 0.1 M glycine buffer, pH 2.8, or pre-incubated in BHI-pH 5.5 for one hour prior to acid-challenge assay **(D)**. Results presented are representatives of three separate sets of experiments with *, **, *** indicating *P* < 0.05, 0.01, and 0.001, respectively, compared to the wild-type (UA159) via Student’s *t*-test.

A markerless frameshift mutant, SppRf, was constructed by PCR-based deletion of cytosine 62 of the *sppR-*coding region. Consistent with our previous study with an allelic exchange of *sppR* (Δ*sppR::Em*) using an erythromycin resistance marker replacing a major portion of the *sppR-*coding sequence (Zeng and Burne, 2019), cytosine 62 deletion and the resulting frameshift mutation had no major effects on the growth phenotypes, including lag phase, doubling time and colony morphology on agar plates, when the resulting frameshift mutant, SppRf, was tested in regular BHI medium. However, when tested in BHI broth adjusted to pH 6.0, strain SppRf had a reduced growth rate (P<0.01) and final optical density (*P* < 0.05) (**Figure 1A**) compared to the parent wild-type UA159. Complementation in SppRf using a wild-type copy of *sppR*, including its cognate promoter, fully restored the phenotype (**Figure 1A**). When growing in BHI supplemented with 25 mM methyl viologen, a common inducer of oxidative stress, the *sppRf* mutant had a drastically extended lag phase, a much reduced growth rate (P<0.05), and lower optical density overnight (P<0.05) when compared to the parent strain under the same conditions (**Figure 1B**). Complementation with *sppR* again fully restored the phenotype in the markerless mutant (**Figure 1B**). When challenged by incubating in a glycine buffer (pH 7.0) containing 58 mM hydrogen peroxide, the survival rate of strain SppRf reduced by >1-log after 60 minutes, and >3-logs after 80 minutes (P<0.001), compared to UA159 (**Figure 1D**). No major differences between the *sppRf* mutant and the wild-type strain UA159 were measured in BHI supplemented with sodium nitroprusside as a source of nitric oxide stress (Ellepola et al., 2024a).

Acid killing assays were used to assess the impact of *sppR* deficiency on acid tolerance response. In acid killing assay by incubation in a glycine buffer of pH 2.8, the survival rate of SppRf and strain UA159 showed no differences following incubation of 30 minutes and 60 minutes. However, when both strains were incubated in BHI broth adjusted to pH 5.5 for one hour before subjected to acid killing assay, the survival rate of strain SppRf was reduced by >1-log after 30 minutes and almost 3-logs after 60 minutes, compared to the parent strain UA159 (P<0.001) (**Figure 1C**). *S. mutans* is known to possess an adaptive acid tolerance response which is featured with enhanced acid resistance following initial exposure to low pH (Lemos et al., 2019). Reduction of acid tolerance following preincubation at pH 5.5 suggest defects of SppRf in adaptive acid tolerance response.

### SppR-deficiency had no major effects on biofilm formation

**Figure 2:**
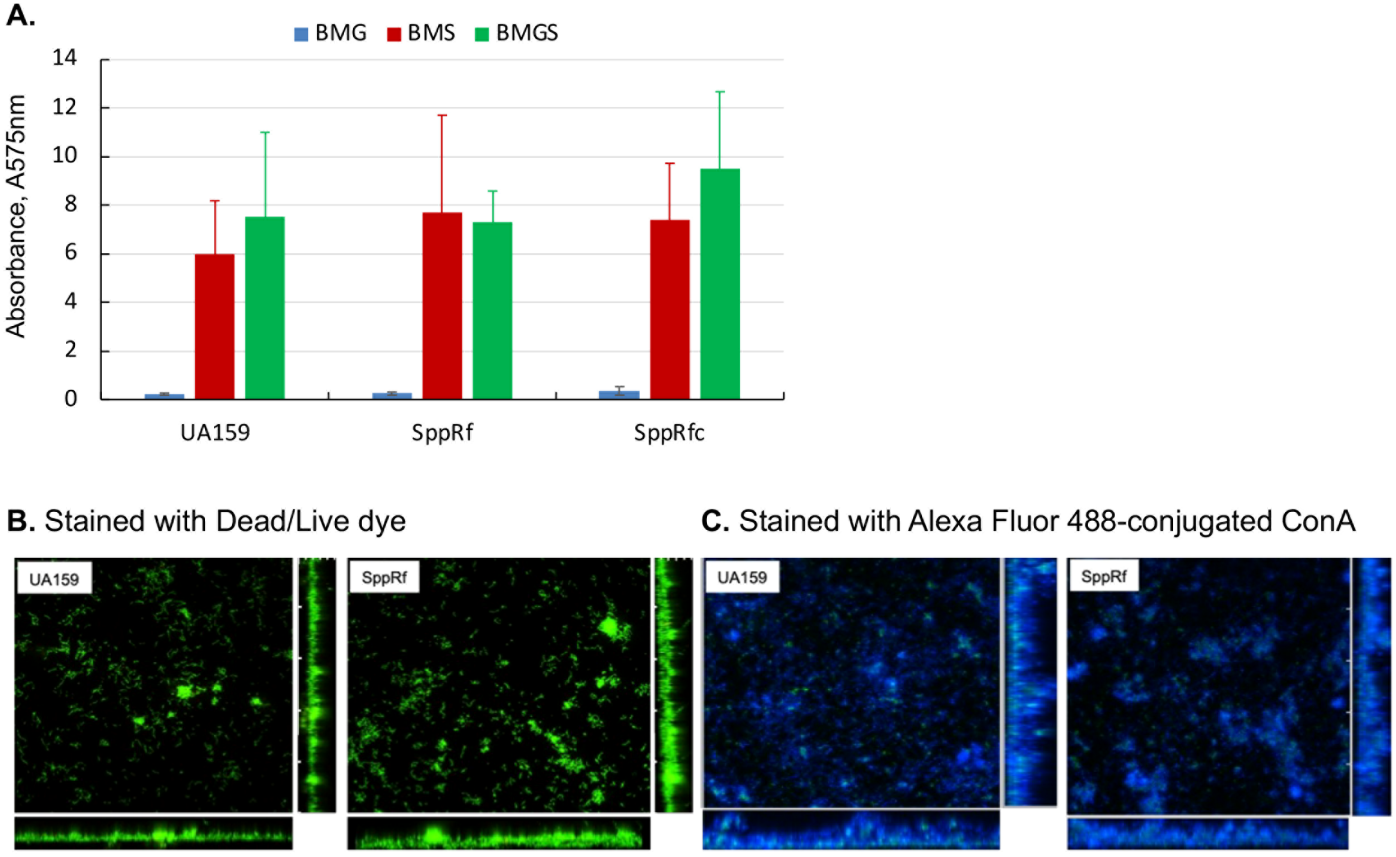
Biofilm formation in 96-well plates **(A)** and on hydroxylapatite disc **(B)**. *S. mutans* strains UA159, SppRf, and SppRf/c were grown in biofilm medium with glucose (BMG), sucrose (BMS), and glucose plus sucrose (BMGS) on 96-well plates and stained with crystal violet **(A)**, or on hydroxylapatite discs and dissected using a confocal laser scanning microscope **(B, C)**. The latter biofilms were stained with Dead/Live staining dye **(B)** and Alexa Fluor 488-conjugated ConA and Hoechst **(C)** prior to confocal analysis. Images presented are representatives of compressed xyz, xz and yz from three different sets of experiments.

When analyzed using a 96-well plate model, the markerless *sppR* mutant and its parent strain showed no major differences in biofilm formation on polystyrene surface in 96-well plates when the bacteria were grown in biofilm medium with and without sucrose (**Figure 2A**). When grown on hydroxyapatite discs, a commonly used model for tooth enamel, and analyzed under a confocal laser scanning microscope with biofilms stained with Dead/Live fluorescent dye (InVitrogen), the *sppR* mutant accumulated similar biovolume of biofilms as the parent strain, UA159 (**Figure 2B**). No major differences were observed in the numbers and their distribution of the membrane-compromised/dead cells. There were no major differences between the mutant and its parent strain when the biofilms were analyzed by Alexa Fluor 488-conjugated ConA (Ex/Em 495/519 nm, ThempFisher Scientific) and Hoeschst 33342 staining, which confers glucose polymers green and the microbial DNA with blue fluorescence (Ex/Em 350/461 nm, ThemoFisher Scientific), respectively (Bitoun et al., 2011; Bitoun et al., 2013) (**Figure 2C**).

### SppR is a positive regulator of *brpA* expression

**Figure 3:**
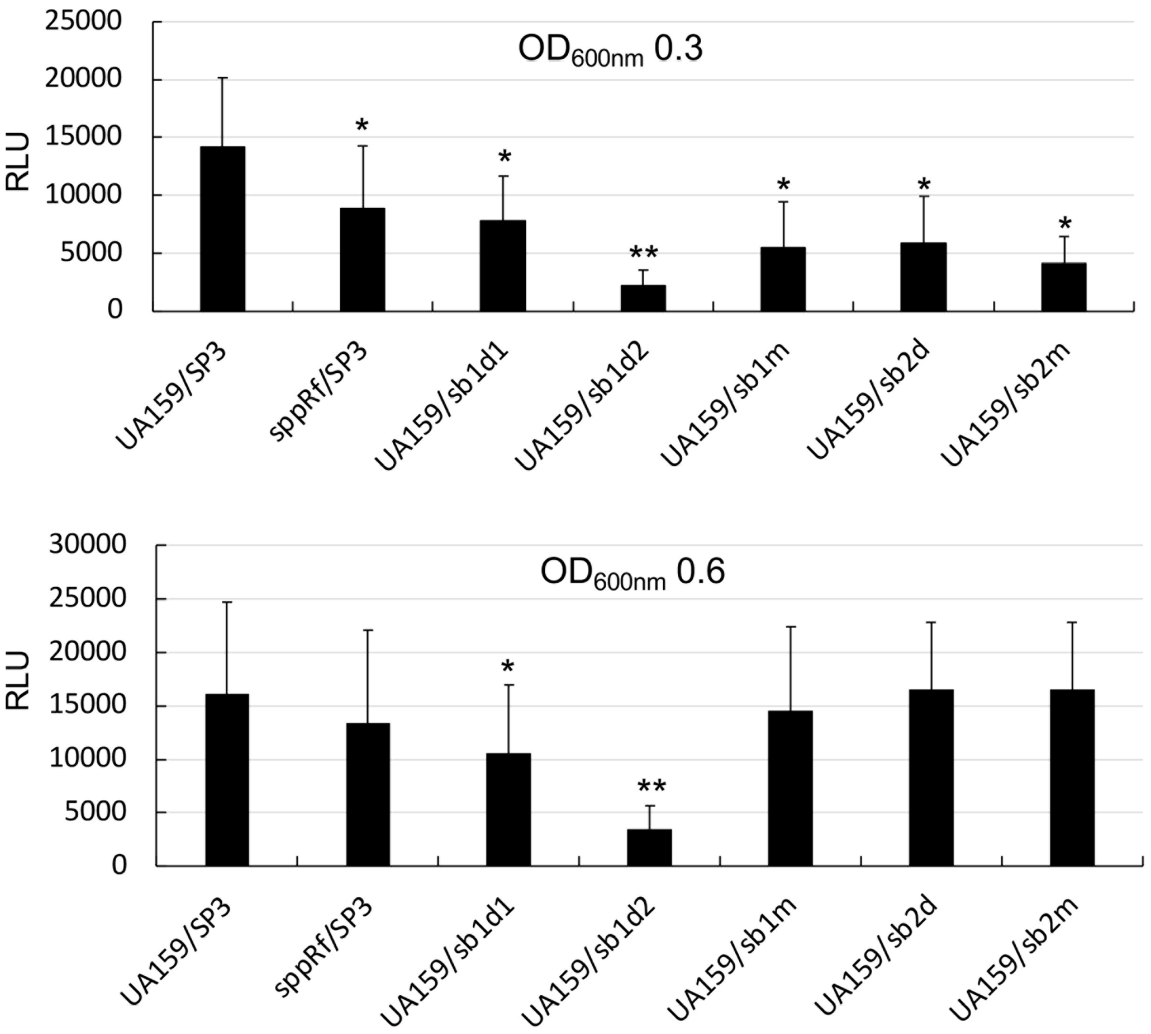
Reporter fusion assays of the *sppR* mutant. *S. mutans* wild-type UA159 and the markerless s*ppRf* mutant carrying the luciferase reporter fusions were grown in BHI broth, and the reporter activities were analyzed during early- (OD_600nm_ of 0.3) and late- (OD_600nm_ of 0.6) exponential phase. Data presented here are expressed as relative light unit normalized with cell density (RLU) with the luciferase reporter being fused to the intact short promoter (SP3), SP3 engineered to contain deletions (sb1d1 and sb1d2) and mutations (sb1m) in SppR-box1, or deletion (sb2d) and mutations (sb2m) in SppR-box2. All experiments were done at least three separate times. * and ** indicate *P* < 0.05 and *P* < 0.01, respectively, compared to SP3 via Student’s *t-*test.

To investigate if SppR plays a direct role in regulating the expression of *brpA*, luciferase reporter fusion assay was employed. When a promoterless luciferase gene was fused to the intact promoter (SP3) of *brpA*, the reporter activity in the *sppR* mutant background was reduced 1.95-fold (P<0.05), compared to the wild-type (**Figure 3**). No such differences were measured when the luciferase reporter was fused to the promoter of lactate dehydrogenase gene as a control. These results suggest that SppR plays the role of a positive regulator in *brpA* expression.

In *B. subtilis*, GlcR binds to a region in the promoter with two triple directed repeats of (ACGCA or TCAAA)-N5-ACTTA-N5-ACTTA (Morabbi Heravi et al., 2019). Two putative GlcR/SppR-binding sites, termed SppR-box1 and SppR-box2, were identified in the intergenic region upstream of *brpA*, with SppR-box1 (AaGatGGAGTAtTTcTTGATgtTTA, low case indicating mismatches to the *Bacillus* GlcR-box) located at nucleotides -34 to -58 relative to translational start codon ATG and SppR-box2 (AaGtgATAGAgCTTgTGATGAtTTt) located at nucleotides -108 to -132 (**Supplementary Figure 1**). Relative to the reporter fusion containing an intact *brpA* promoter (SP3), internal deletion of nucleotides GATgtTTA of SppR-box1 (sb1d1, **Figure 3**) led to a reduction of 1.95-fold (P<0.05), whereas deletions of the conserved AaGat and TTcTT of box1 (sb1d2, **Figure 3**) led to a reduction of reporter activity by 7.7-fold (*P* < 0.001). Site-directed mutations of these regions from AaGatGGAGTAtTTcTTGATgtTTA to tatatGGAGTttaacTTGATgtaat (sb1m1, **Figure 3**) also caused reduction of reporter activity by 3.55-fold (P<0.01). Similar results were observed with deletion and mutations of the SppR-box2, including deletion of AaGtgATAGATGATG (sb2d1, **Figure 3**) and mutations from ACgCTGTT to taGCTaaa (sb2m1, **Figure 3**). Interestingly, no major effects were observed with SppR-box 1 mutations (sb1m1) and the deletion and mutations of SppR-box2 (sb2d1 and sb2m1) in late exponential phase (OD_600nm_ ~0.6) cultures. These results further suggest that SppR acts as a positive regulator of *brpA*, by directly interacting with its cognate elements conserved upstream of BrpA-coding sequence; and its function seems to be limited to actively growing cells. The results that deletions of the SppR-box1 led to 7.7-fold reduction, while SppR deficiency alone had only 1.95-fold differences also suggest that other positive regulator(s) that interact with regions that partly overlap the SppR-box1 are likely functional in *brpA* expression.

### Recombinant SppR binds to the *brpA* promoter *in vitro*

**Figure 4:**
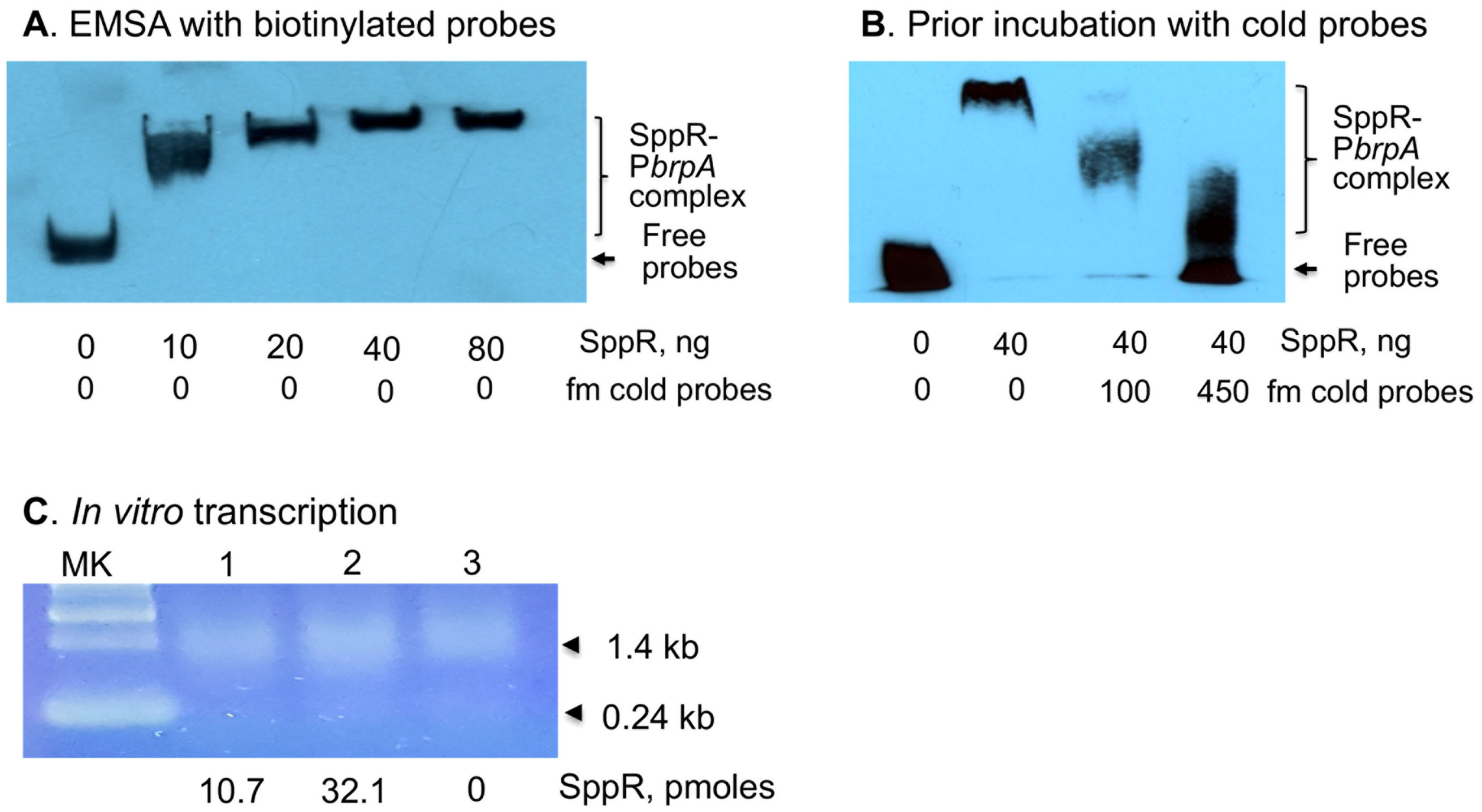
EMSA **(A, B)** and IVT **(C)** analysis. In EMSA, inclusion of recombinant protein rSppR resulted in mobility shift of the biotinylated *brpA* promoter probe and such effects were concentration dependent **(A)**. Prior incubation with un-biotinylated *brpA* probe dismantled such a SppR-dependent shift **(B)**. In IVT, inclusion of rSppR (lanes 1&2) enhanced *brpA* transcription, compared to control that received make-up buffer (lane 3). Data presented are representatives of more than three independent experiments.

A recombinant SppR protein (rSppR) with a His-tag at its N-terminus was overexpressed in *E. coli* M15, purified, and then used in EMSA to further assess if SppR directly interacts with the *brpA* promoter. The results showed that addition of rSppR retarded the electrophoretic mobility of a biotinylated DNA probe containing the *brpA* promoter in a concentration-dependent manner (**Figure 4A**). Furthermore, prior incubation of rSppR with an identical yet un-biotinylated promoter probe was shown to significantly reduce such electrophoretic shift (**Figure 4B**). These results demonstrated the ability of SppR to directly interact with the *brpA* promoter, whereby influencing the expression of *brpA* gene.

### Recombinant SppR enhances *brpA* transcription *in vitro*

To further assess how SppR could regulate the transcription of *brpA*, IVT assays were carried out using the PCR amplicon of the full-length *brpA*, including the promoter region, as the template. Gel electrophoresis of the reaction detected two transcripts of *brpA*, with one, at ~1.4 kb, being the expected full-length mRNA and the other, at ~0.24 kb, a likely product of pre-mature termination (**Figure 4C**). The abundance of the full-length transcript was significantly enhanced when 32.1 pmoles (1.28 µM, final concentration) of rSppR was added to the reaction. In contrast to XRE, a recently identified negative regulator of *brpA* (Wen et al., 2026), inclusion of rSppR reduced the intensity of the 0.24 kb transcript, a likely product of transcription termination.

### The SppR mutant has an altered transcriptomic profile

RNA-seq analysis was used to delineate the scope as well as the underlying mechanisms of SppR-mediated regulation in *S. mutans* pathophysiology. This was done using the allelic exchange mutant with wild-type UA159 as the control when they were growing in regular BHI broth until mid-exponential phase (OD_600nm_ ~0.4). The results showed that deletion of *sppR* resulted in some major changes of the transcriptomic profile, as indicated by the Heatmap (**Supplementary Figure 3**) and the list of differentially transcribed genes (**Table 2**). When compared to the parent strain UA159, sixty-eight genes were down-regulated and sixty-eight were up-regulated by >2-fold (*P<*0.05) in the *sppR* mutant. Among the up-regulated genes were those for nucleic acid metabolism and DNA repair (e.g., dihydroorotate oxidase and A/G-specific adenine glycosidase MutY), protein secretion (SecE and SecY), cell envelope biogenesis and cell division (e.g., phosphor-N-acetylmuramoyl-pentapetide-transferase MraY), and sugar and central metabolism (e.g., antiterminator LicT, and PTS transporter EIIC). Of the down-regulated genes, several are for galactose- (e.g., galactoside permease LacB and LacF, galactose-6-phosphate kinase, and galactokinase), mannose/fructose- (EIIB^Man^), and mannitol- (EIIBmannitol) metabolism. The down-regulated genes also included those for acid tolerance response (Malolactic enzyme), DNA metabolism and repair (e.g., class I SAM-dependent methyltransferase SMT, DNA processing protein DprA), environmental stress tolerance response LytTR family transcriptional regulator HdrR (Xie et al., 2020), mutanobactin A biosynthesis and transport (MubI/J/M/P/Z), and DNA uptake and competence development (ComGF/E/D/C/B/A) (Petersen et al., 2005; Chen et al., 2006). Eighteen genes, 5 up-regulated and 13 down-regulated, were found to code for hypothetical proteins with unknown functions. Of note, the *comG* genes and those for the mutanobactin synthesis and transport are arranged in apparent operons (SEED Viewer v2.0, https://rast.nmpdr.org/seedviewer.cgi?page=Home). It is also noteworthy that among the down-regulated genes were *sppA*, which is known to be co-transcribed with *sppR* as a bicistronic operon (Zeng and Burne, 2019). Given the negative regulation exerted by SppR on this operon (Zeng and Burne, 2019), down-regulation of *sppA* in the allelic exchange *sppR* mutant suggests a likely polar effect of the antibiotic resistance marker on the down-stream *sppA.*

**Table 2:** Up- and down-regulated genes in response to sppR deficiency*.

		Up-regulated genes in response to *sppR* deficiency		
NCBI Locus tag	Gene name/ GenBank ID	Description	Fold Change (sppR/wildtype)	P Value
SMU_RS02835	pyrD	dihydroorotate oxidase	6.517676068	3.01E-09
SMU_RS03570		IS3-like element ISSmu1 family transposase	5.702970922	0.0174753
SMU_RS03000	SMU_635	VIT1/CCC1 transporter family protein	5.129250943	1.62E-08
SMU_RS05795	SMU_1258c	restriction endonuclease	4.772676238	0.014533
SMU_RS04500	licT	BglG family transcription antiterminator LicT	4.540486036	2E-08
SMU_RS00485	SMU_87	TIGR01440 family protein	4.532348025	6.89E-07
SMU_RS10070		hypothetical protein	4.212002361	0.0001898
SMU_RS10140	SMU_1395c	hypothetical protein	4.122481583	0.0000276
SMU_RS07370	SMU_1623c	CvfB family protein	3.882604574	5.84E-08
SMU_RS01455	SMU_293	HXXEE domain-containing protein	3.402689364	2.94E-06
SMU_RS02610	SMU_543	rhodanese-like domain-containing protein	3.392946901	3.01E-08
SMU_RS06360	gbpC	glucan-binding protein GbpC	3.390821157	0.0000779
SMU_RS10050		type II toxin-antitoxin system PemK/MazF family toxin	3.220339676	0.0050493
SMU_RS05420	atmA	amino acid ABC transporter substrate-binding protein	3.030976357	7.57E-07
SMU_RS07420	SMU_1634c	NUDIX hydrolase	2.99759508	5.33E-08
SMU_RS03930	SMU_848	ribosomal-processing cysteine protease Prp	2.953731454	2.43E-06
SMU_RS02620	SMU_547	DUF3165 family protein	2.944795104	2.84E-07
SMU_RS03935	rpmA	50S ribosomal protein L27	2.930797912	4.89E-06
SMU_RS04620	gtfB	glucosyltransferase GtfB	2.831675777	0.000055
SMU_RS00875	SMU_177	PTS sugar transporter subunit IIC	2.828492395	0.0218822
SMU_RS06075	SMU_1319c	esterase family protein	2.804115998	0.0001486
SMU_RS04595	fatB	siderophore ABC transporter substrate-binding protein	2.764199141	1.89E-07
SMU_RS02200	SMU_457	hypothetical protein	2.728317705	1.04E-06
SMU_RS06905	glnP	amino acid ABC transporter permease	2.652341441	6.22E-06
SMU_RS09155	secY	preprotein translocase subunit SecY	2.63392642	3.54E-06
SMU_RS05875	ezrA	septation ring formation regulator EzrA	2.616165902	0.0000426
SMU_RS05755	SMU_1249c	DUF4352 domain-containing protein	2.603161555	0.0001311
SMU_RS05740	SMU_1245c	YjjG family noncanonical pyrimidine nucleotidase	2.582708789	4.12E-06
SMU_RS08275	SMU_1803c	DUF4230 domain-containing protein	2.563435893	0.0001932
SMU_RS06410	#N/A	IS3-like element ISSmu1 family transposase	2.55652237	0.0056069
SMU_RS08845	SMU_1946	DUF4956 domain-containing protein	2.519493878	2.15E-07
SMU_RS04705	pycB	oxaloacetate decarboxylase subunit alpha	2.507001925	3.4E-07
SMU_RS02355	pflC	glycyl-radical enzyme activating protein	2.486639553	7.46E-06
SMU_RS04735		cysteine-rich KTR domain-containing protein	2.477403516	0.0000215
SMU_RS04730	SMU_1028	alpha/beta fold hydrolase	2.47581193	0.0000216
SMU_RS08215	SMU_1789c	YebC/PmpR family DNA-binding transcriptional regulator	2.46446479	0.0000168
SMU_RS04495	potD	ABC transporter substrate-binding protein	2.401904422	0.0000225
SMU_RS02550	pheA	chorismate mutase	2.37274018	0.0014528
SMU_RS04960	SMU_1076	ECF transporter S component	2.371082935	0.0000421
SMU_RS00435	ampD	glycoside hydrolase family 73 protein	2.369380372	0.0000291
SMU_RS02975	SMU_630	serine hydrolase	2.325012676	5.39E-06
SMU_RS03765	mutT	8-oxo-dGTP diphosphatase	2.314003655	0.0153988
SMU_RS02545	SMU_530c	DUF975 family protein	2.309386468	0.000649
SMU_RS02195	mraY	phospho-N-acetylmuramoyl-pentapeptide- transferase	2.301705218	0.0001252
SMU_RS07015	SMU_1546	GtrA family protein	2.223312441	0.0000139
SMU_RS06620	rmlB	dTDP-glucose 4,6-dehydratase	2.205399858	1.85E-06
SMU_RS00510	pyrG	CTP synthase	2.181863474	0.0000467
SMU_RS08540	mutY	A/G-specific adenine glycosylase	2.181174881	0.0000141
SMU_RS06055	SMU_1315c	ABC transporter ATP-binding protein	2.179295448	0.0004074
SMU_RS05780	SMU_1254	HAD family hydrolase	2.170256666	0.0000612
SMU_RS04215	SMU_911c	DUF308 domain-containing protein	2.155658255	0.0012083
SMU_RS03350	SMU_711	DUF4430 domain-containing protein	2.152657618	0.0008752
SMU_RS06290	leuD	3-isopropylmalate dehydratase small subunit	2.141328137	0.0046904
SMU_RS08980	SMU_1975c	type II CAAX endopeptidase family protein	2.13233796	0.000069
SMU_RS01415	SMU_283	Blp family class II bacteriocin	2.128827916	0.0002364
SMU_RS05800	SMU_1259	Eco57I restriction-modification methylase domain-containing protein	2.126193776	0.0107887
SMU_RS02585	trpA	tryptophan synthase subunit alpha	2.122924811	0.0001329
SMU_RS00585	tagR	MurR/RpiR family transcriptional regulator	2.113062489	0.0016532
SMU_RS09040	SMU_1988c	DUF1033 family protein	2.095954666	0.0017219
SMU_RS07045	SMU_1553c	hypothetical protein	2.084360684	0.0034741
SMU_RS04365	pnuC	nicotinamide riboside transporter PnuC	2.082770473	0.0003093
SMU_RS05760	SMU_1250c	hypothetical protein	2.081227825	0.023879
SMU_RS08250	SMU_1798c	bis(5'-nucleosyl)-tetraphosphatase (symmetrical) YqeK	2.050281585	0.0000838
SMU_RS08855	secE	preprotein translocase subunit SecE	2.049440829	0.0045892
SMU_RS07655	Orf11	DUF1803 domain-containing protein	2.04226229	0.0025551
SMU_RS07395	SMU_1628	DUF3397 domain-containing protein	2.040152232	0.0004909
SMU_RS05135	SMU_1116c	hypothetical protein	2.029621366	0.0003213
SMU_RS08645	SMU_1895c	Blp family class II bacteriocin	2.003412902	0.0002764

Down-regulated genes in response to *sppR* deficiency				
NCBI Locus tag	Gene name/ Genbank tag	Description	Fold change (wildtype/sppR)	P Value
SMU_RS00420	SMU_73	PFL family protein	2.912308191	0.024670011
SMU_RS00525	nigB	PTS system mannose/fructose/N-acetylgalactosamine-transporter subunit IIB	2.72686821	0.000190661
SMU_RS00530	nigC	PTS mannose/fructose/sorbose/N-acetylgalactosamine transporter subunit IIC	2.043888546	0.00348028
SMU_RS00700	mleS	malolactic enzyme	4.23334872	0.0000218
SMU_RS00705	mleP	AEC family transporter	4.659144438	0.000115112
SMU_RS00710	oxdC	oxalate decarboxylase family bicupin	4.736293516	0.000049
SMU_RS00715	gshR	dihydrolipoyl dehydrogenase family protein	4.17760084	0.0000868
SMU_RS00720	SMU_141	YoaK family protein	3.306865156	0.0000684
SMU_RS00885	SMU_179	NADPH-dependent FMN reductase	2.155070695	0.000250201
SMU_RS01020	SMU_194c	ASCH/PUA domain-containing protein	2.279905422	0.038852302
SMU_RS01025	SMU_195c	hypothetical protein	2.379539663	0.036603804
SMU_RS01030	SMU_196c	phage tail tip lysozyme	2.71045804	0.014087832
SMU_RS01035	SMU_197c	hypothetical protein	2.916460527	0.012879101
SMU_RS01040	tpn	ATP-binding protein	2.535828164	0.029861114
SMU_RS01045	SMU_199c	conjugal transfer protein	3.394894512	0.007100065
SMU_RS01055	SMU_201c	conjugal transfer protein	2.901983732	0.030353664
SMU_RS01065	SMU_205c	hypothetical protein	2.253199023	0.044609921
SMU_RS01075	SMU_207c	replication initiation factor domain-containing protein	2.530628803	0.019897448
SMU_RS01080	SMU.208c	FtsK/SpoIIIE domain-containing protein	2.684483624	0.005738901
SMU_RS01085	SMU_209c	DUF961 family protein	2.353168433	0.0179913
SMU_RS01090	SMU_210c	hypothetical protein	2.557876476	0.011608806
SMU_RS02390	comFA	DEAD/DEAH box helicase	10.62084841	0.000000227
SMU_RS02440	sppR	sugar-phosphate phosphatase transcriptional regulator SppR	4.397193115	0.00000743
SMU_RS02445	sppA	fructose-phosphate phosphohydrolase SppA	12.91001285	4.86E-09
SMU_RS02895	bsp	SH3 domain-containing protein	2.474516014	0.001825325
SMU_RS02950	comEA	helix-hairpin-helix domain-containing protein	7.15533629	0.000000205
SMU_RS02955	comEC	DNA internalization-related competence protein ComEC/Rec2	3.611469409	0.005341179
SMU_RS03145	argJ	bifunctional ornithine acetyltransferase/N-acetylglutamate synthase	2.766580423	0.026312243
SMU_RS03875	lytF	GBS Bsp-like repeat-containing protein	2.127169439	0.000271072
SMU_RS04060	tpn	IS3 family transposase	6.648952334	0.005873866
SMU_RS04115	galK	galactokinase	2.122605516	0.001799544
SMU_RS04510		prolyl oligopeptidase family serine peptidase	21.46139246	0.002355438
SMU_RS04605	dprA	DNA-processing protein DprA	8.504707544	0.0000014
SMU_RS05020	apbE	FAD:protein FMN transferase	2.103781679	0.002826516
SMU_RS05455	mtlA	PTS mannitol transporter subunit IICB	3.720231012	0.007608827
SMU_RS05825	hisF	imidazole glycerol phosphate synthase subunit HisF	5.363007054	0.018769869
SMU_RS05840	SMU_1267c	YrdB family protein	14.18804468	0.000792146
SMU_RS06125		IS982 family transposase	3.289920726	0.001710014
SMU_RS06130	mubP	mutanobactin A biosynthesis phosphopantetheinyl transferase MubP	9.722465235	0.000341718
SMU_RS06135	mubJ	mutanobactin A biosynthesis reductase MubJ	10.26938036	0.00021992
SMU_RS06140	mubI	mutanobactin A biosynthesis transacylase MubI	13.03238623	0.000115888
SMU_RS06145	mubM	mutanobactin A biosynthesis alpha/beta hydrolase MubM	17.97975258	0.0000627
SMU_RS06150	mubZ	mutanobactin A system MFS transporter MubZ	23.21530671	0.0000435
SMU_RS06245	SMU_1367c	class I SAM-dependent methyltransferase	21.52211054	0.000000791
SMU_RS06485	pdhD	dihydrolipoyl dehydrogenase	7.132071751	0.000978738
SMU_RS06490	clpB	ATP-dependent Clp protease ATP-binding subunit	4.843806982	0.000428727
SMU_RS06775	lacF	PTS lactose transporter subunit IIA	2.071306035	0.027101999
SMU_RS06780	lacD	tagatose-bisphosphate aldolase	12.42767542	0.002596757
SMU_RS06785	lacC	tagatose-6-phosphate kinase	2.201356724	0.001706715
SMU_RS06790	lacB	galactose-6-phosphate isomerase subunit LacB	2.054098684	0.002171205
SMU_RS06795	lacA	galactose-6-phosphate isomerase subunit LacA	2.169778431	0.004188146
SMU_RS07320	SMU_1613c	dephospho-CoA kinase	2.661726113	0.035093134
SMU_RS08020	cas2	CRISPR-associated endonuclease Cas2	2.015789199	0.000757315
SMU_RS08025	cas1c	type I-C CRISPR-associated endonuclease Cas1c	2.166525252	0.000178144
SMU_RS08030	cas4	CRISPR-associated protein Cas4	2.486729006	0.0000717
SMU_RS08035	cas7c	type I-C CRISPR-associated protein Cas7/Csd2	2.732012378	0.00000541
SMU_RS08040	cas8c	type I-C CRISPR-associated protein Cas8c/Csd1	2.538951005	0.00000803
SMU_RS08045	cas5c	type I-C CRISPR-associated protein Cas5c	2.484128598	0.00000838
SMU_RS08050		CRISPR-associated helicase/endonuclease Cas3	2.088850228	0.0000358
SMU_RS08500	hdrR	LytTR family transcriptional regulator HdrR	2.031219279	0.009620579
SMU_RS09005	SMU_1980c	competence type IV pilus minor pilin ComGG	8.491187147	0.00000233
SMU_RS09010	comGF	competence type IV pilus minor pilin ComGF	7.362973479	0.00000151
SMU_RS09015	SMU_1982c	competence type IV pilus minor pilin ComGE	7.778925883	0.00000224
SMU_RS09020	comYD	competence type IV pilus minor pilin ComGD	8.519307008	0.000000106
SMU_RS09025	comYC	competence type IV pilus major pilin ComGC	7.297182234	0.000000183
SMU_RS09030	comYB	competence type IV pilus assembly protein ComGB	9.761936918	0.000000122
SMU_RS09035	comYA	competence type IV pilus ATPase ComGA	4.676609663	0.00000531
SMU_RS09720	gabD	NAD-dependent succinate-semialdehyde dehydrogenase	2.10092866	0.002889275

*Results were expressed as transcript ratios in fold-change.

## Discussion

The results presented here have demonstrated that SppR (formerly GlcR) is a transcriptional regulator with a significant role in *S. mutans* physiology, including acid and oxidative stress tolerance response. As compared to the wild-type, SppR-deficiency led to major reduction in the ability to survive low pH and oxidative stressors, and such effect appears to be specifically related to adaptive acid tolerance response (ATR). At the transcriptomic level, SppR deficiency caused some major alterations in gene regulation, including those related to acid and oxidative stress tolerance response. Luciferase reporter fusion assays, along with EMSA and *in vitro* transcription assay confirmed that SppR is a positive regulator of BrpA, a surface-associated multi-function protein with critical role in regulating cell envelope biogenesis.

Our recent studies have shown *sppR* is co-transcribed with its downstream *sppA* as a bicistronic operon, and SppR is an autoregulator that represses the transcription of *sppA* (Zeng and Burne, 2019). *sppA* encodes a fructose-1-phosphatase that plays an important role in regulation of sugar phosphate stress in *S. mutans.* Accumulation of fructose-1-phosphate, as a result of active growth with fructose as the major carbon and energy source, not only induces de-repression of *sppA* expression, but also significantly altered biofilm maturation through increased cell lysis and eDNA release. Constitutive expression of SppA via a plasmid or deletion of *sppR* alleviate sugar-phosphate stress and reverse the fructose effects on biofilms (Zeng and Burne, 2019). Coincidently, our RNA-seq analysis showed a major reduction of *sppA* transcript in the allelic exchange *sppR* mutant. This is likely a result of polar effect of the erythromycin resistance marker that was used to replace a large portion of the *sppR-*coding sequence (Wen and Burne, unpublished). To eliminate the polar effects, a markerless *sppR* mutant was constructed and used for further studies of SppR in *S. mutans* fitness and biofilm formation. Of note, no major differences were observed when the deletional mutant was compared to the frameshift mutant in growth characteristics in regular BHI broth and biofilm medium; and both displayed some major effects when the mutants were challenged with low pH and oxidative stressor methyl viologen and hydrogen peroxide, resulting in a reduced growth and weakened tolerance, as compared to the wild-type. Different from the frameshift mutant, however, complementation assay of the allelic exchange mutant with the wild-type copy of *sppR* plus its cognate promoter failed to fully restore the phenotype when growing in the presence of methyl viologen. This discrepancy can also be in part attributed to the reduced expression of *sppA*, as a result of polar effect of the allelic exchange mutagenesis.

*S. mutans* is known for its ability to survive low pH in the oral cavity and become more resistant to lower pH following initial exposure to low pH, a phenomenon called adaptive acid tolerance response (Lemos and Burne, 2008; Lemos et al., 2019). Adaptive acid tolerance response is often featured with elevated expression of proton pump F_1_F_0_-ATPase, molecular chaperones such as DnaK and GroEL, and DNA repair system in response to low pH exposure. The results presented here showed that *sppR* is involved in regulation of acid tolerance response, and such effects are mostly related to the adaptive acid tolerance response. From RNA-seq analysis, *sppR* deficiency led to altered expressions of several genes known to play important roles in acid tolerance response and DNA repair, which include down-regulation of the malolactic enzyme (Mle), DNA processing protein DprA and SAM-dependent methyltransferase (SMT). A key component of the acid tolerance system, Mle enzyme converts malic acid (malic acid as an abundant, strong acid in foods such as fruit juice) to lactic acid and CO_2_, reducing intracellular malate accumulation and helping maintain pH homeostasis (Sheng et al., 2010). DprA is crucial for natural transformation but also plays a significant role in DNA repair and bacterial virulence (38). SMT is known for its role in methylation of DNA and proteins, a process that affects gene expression, survival, and other cellular functions (Gao et al., 2023). Thus, down regulation of Mle enzyme, DprA, and SMT likely have a major impact on the ability of the *sppR* mutant to adapt to and survive low pH. Of the up-regulated gene, MutY is an A/G-specific adenine DNA glycosylase in *Escherichia coli* that plays a key role in base excision repair and mismatch repair. Up-regulation of MutY also indicates that the *sppR* mutant is likely suffering from DNA damage induced from low pH and oxidative stressors such as hydrogen peroxide in the growth environment.

Multiple factors regulate oxidative stress tolerance response in *S. mutans*, including Rex, SpxA1, SloR, and PerR (Bitoun et al., 2012b; Kajfasz et al., 2015; Lemos et al., 2019; Ellepola et al., 2021; Ruxin et al., 2021); and the pathways in oxidative stress tolerance response such as the molecular chaperones and DNA repair partly overlap the ones in acid tolerance response (Lemos and Burne, 2008; Lemos et al., 2019). Our recent study on fructose-mediated stress response in *S. mutans* identified a core of stress genes shared by fructose, hydrogen peroxide, and in part by acidic pH (Walker et al., 2025). Results presented herein also substantiated the theory that SppR plays a direct role in regulation of oxidative stress tolerance response; and its deficiency caused some major defects in the ability to survive oxidative stressors like hydrogen peroxide and superoxide induced by methyl viologen. Among others, down regulation of genes involved in DNA repair, such as DprA and SMT are likely part of the underlying factors. In addition, down-regulation of the *mubPJIMZ*, genes for biosynthesis and transporter of mutanobactin, a non-ribosomal lipopeptide with a major role in response to oxygen and oxidative stress (Wu et al., 2010), also likely contributed to the reduced oxidative stress tolerance response of the *sppR* mutant.

*S. mutans* possesses multiple pathways to adhere to and persist on the tooth surface, including the sucrose-dependent pathways involving glucosyltransferases and glucan-binding proteins (Burne, 1998; Lemos et al., 2019). The glucosyltransferases catalyze the production of adhesive polymers that coat the tooth and microbial surfaces, while glucan-binding proteins bind to the glucans facilitating surface and inter-cellular adherence and accumulation of multi-cellular clusters. Notably, *gtfB* for glucosyltransferase GtfB and *gbpC* for glucan-binding protein GbpC were also in the list of up-regulated genes. RealTime-qPCR was used to further evaluate the differential transcription of *gbpC* and *gtfB*, and the results showed a 1.56-fold increase in copy numbers of *gtfB* (9.63E + 8 for the wildtype vs 1.51E + 9 for the *sppR* mutant) and 2.88-fold increase of *gbpC* (2.14E + 9 for UA159 vs 6.17E + 9 for the *sppR* mutant). However, neither the 96-well plate model via crystal violet staining nor the hydroxylapatite disc model under the microscope showed any significant increases in biofilm formation by the *sppR* mutant under the conditions tested (data not shown). Biofilm formation is highly regulated in response to environmental cues, and various factors including molecular chaperones, multiple two-component regulatory systems and BrpA play a role in *S. mutans* biofilm formation. As a positive regulator, *sppR* deficiency will reduce *brpA* expression, contributing to the reduced biofilm formation. In addition, the *sppR* mutant is suffering with some major defects in acid and especially oxidative stress tolerance. Therefore, it is not surprising that despite some significant increases of *gtfB* and *gbpC* transcription, the *sppR* mutant did not show any significant changes in biofilm formation.

It is apparent that *sppR* deficiency affects the expression of a large number of genes, including those with major roles in sugar metabolism, stress tolerance response, DNA repair, *brpA* expression and biofilm formation. It is also worth noting that the altered expression of certain genes identified by RNA-seq in the allelic exchange mutant, including those related to oxidative stress tolerance response and sugar metabolism, could be partly attributable to the polar effect on *sppA* (Zeng and Burne, 2019). BHI medium is expected to contain a very small amount of contaminating fructose. At first glance, reduced expression of *sppA* may lead to phosphosugar stress under fructose conditions, due to accumulation of F-1-P (Zeng and Burne, 2019). However, given the low basal-level expression of the *sppRA* operon and the high intracellular F-1-P levels required for inducing the operon, the impact of the polar effect should be very minor. In fact, measurement of F-1-P in an *sppA* deletion mutant showed no substantial change, even under 20 mM fructose, comprared to the wild-type parent (Zeng and Burne, 2019). This supposition was further supported by the strong, overlapping phenotype of both *sppR* mutants under oxidative conditions. Nonetheless, the frameshift *sppR* mutant SppRf represents an *sppR*^-^*sppA*^+^ state, as opposed to the *sppR*^-^*sppA*^-^ state in the allelic exchange mutant, and plans are in place to further investigate the impact of loss of SppR or SppA, together with fructose content.

The role of SppR as a positive regulator in *brpA* expression is further supported by EMSA assay and *in vitro* transcription assay. Of note, RealTime-qPCR was also used to analyze the expression of *brpA*, and the results showed an average of 1.56-fold reduction in copy numbers when the *sppR* mutant was compared to its parent strain, UA159. In addition, complementation of the *sppR* mutant with the wild-type *sppR* under a constitutive promoter in pIB184 increased the level of *brpA* transcription by 2.49-fold, compared to the control that carried the empty vector pIB184 (*P* < 0.05) (Biswas et al., 2008). Relative to the wild-type UA159, the wild-type carrying the extra-copies of *sppR* in pIB184 also had 3.08-fold increases in *brpA* transcripts (*P* < 0.05). Obviously, this is different from the result of RNA-seq, which did not identify *brpA* as differentially expressed in response to *sppR* deficiency. We believe such a discrepancy can be in part attributed to the relatively low transcript level of *brpA* (Wen and Burne, 2004). Unlike RealTime-qPCR that is highly sensitive and especially fit for low-abundance transcripts, RNA-seq only provides a snapshot of the broad transcriptome and is prone to biases for genes in abundance.

GlcR in *B. subtilis* was shown to bind two triple directed repeats of (ACGCA or TCAAA)-N5-ACTTA-N5-ACTTA in the promoter region of target genes (Morabbi Heravi et al., 2019). Two putative binding sites, termed SppR-box1 and SppR-box2, were identified in the *brpA* promoter region, and PCR-based deletions and site-directed mutagenesis analysis showed that both may play a role in SppR-mediated regulation of *brpA* expression. The reporter fusion results also suggest that SppR is likely associated with a regulatory complex that may also interact with the SppR-box1 and/or the flanking region in regulation of *brpA* expression. What other factors are involved and how they interact with each other and the promoter region await further investigation.

BrpA and member of the LytR-CpsA-Psr family proteins are key enzymes of the cell envelop biogenesis, including attachment of the cell wall-associated rhamnose glucose polymer (RGP) in *S. mutans* (De et al., 2017). RGP synthesis is initiated by uridine diphosphate (UDP)- *N-*acetylglucosamine-1-phosphate (GlcNAc) transferase RgpG, which links GlcNAc onto a membrane-anchored bactoprenol lipid carrier (Yamashita et al., 1999), and the poly-rhamnose backbone is elongated via specific rhamnosyltransferases and further modified with glucose sidechains via glucosyltransferases (Kovacs et al., 2019). It is therefore not surprising that the expression of *brpA* is significantly influenced by SppR and sugar metabolism. In fact, multiple factors, including LevR (response regulator of the four-component LevQRST system in fructose/mannose metabolism) and NagR (regulator of amino sugar metabolism), are shown to play a role in *brpA* expression (Liao et al., 2017). If and how SppR and other regulators interact and to what environmental cues they respond in regulation of *brpA* expression await further investigation.

The results presented here have shown that SppR functions at the interface between carbohydrate metabolism and stress adaptation in *S. mutans*, providing an important regulatory link between nutrient availability, intracellular metabolic balance, and envelope biogenesis/homeostasis. As a transcriptional repressor of the *spp* operon, SppR limits expression of phosphosugar-degrading enzymes under basal conditions. During growth on PTS-transported carbohydrates, particularly fructose, excess intracellular sugar-phosphates accumulate, alleviating SppR repression and enabling rapid restoration of Fructose-1-Phosphate homeostasis. This regulatory mechanism allows *S. mutans* to efficiently exploit diverse sugars at micro-concentrations while avoiding sugar-phosphate stress, a condition that can impair growth, disrupt redox balance, and compromise cell envelope homeostasis. In this context, SppR-controlled metabolic homeostasis likely converges with envelope-focused regulatory systems, including BrpA. By ensuring balanced carbohydrate uptake and utilization, SppR may indirectly support BrpA-dependent attachment of RGP to the peptidoglycan, maintenance of envelope integrity, and resistance to envelope stressors. Together, SppR-mediated metabolic regulation and BrpA-regulated cell envelop biogenesis/homeostasis form a coordinated network that integrates nutrient sensing, stress tolerance, and pathogenic fitness in *S. mutans*. We have reported previously that fructose metabolism led to bacterial autolysis, release of eDNA, and increased biofilm formation (Zeng and Burne, 2019); phenotypes consistent with change in envelope homeostasis. How exactly the SppR-mediated metabolic regulation and BrpA-regulated cell envelope biogenesis/homeostasis are coordinated in response to environmental cues await further investigation. It is also worth noting that while *sppR* is autoregulated, the expression of *sppR* is primarily induced by the relatively high levels of fructose (Zeng and Burne, 2019), which is most likely mediated by Fructose-1-Phosphate. On the other hand, neither oxidative stressor hydrogen peroxide and low pH was shown to have any major impact on *sppR* expression (Baker et al., 2015; Kajfasz et al., 2017). The exact scope and underlying mechanisms of the SppR-mediated regulation in *S. mutans* pathophysiology also await further studies under more relevant conditions.

## Data Availability

The datasets presented in this study can be found in online repositories. The names of the repository/repositories and accession number(s) can be found below: https://www.ncbi.nlm.nih.gov/, GSE328150.
