# Supplementary material for: SppR in Streptococcus mutans coordinates stress tolerance response and BrpA expression
Source: Front Cell Infect Microbiol. 2026 Jul 27;16:1907905. doi: 10.3389/fcimb.2026.1907905 (PMC13454231; doi:10.3389/fcimb.2026.1907905)
Supplement: Supplementary file 1 [file SupplementaryFile1.pdf]

Table S1. Primers used in this study

Name	Oligonucleotide sequence (5'-3')	Application
sppR55'	aagaattccgactcgaccttgccc	*5' fragment for sppR deeltion*
sppR53'	atgagctctagttgtccttgcctatcaagc	
sppR35'	ataagctttgttctcaagctggctcagc	*3' fragment for sppR deeltion*
sppR33'	ttgcatgctcagtaatcgtatcacgc	
sppRe5Kp	atggtacccaagaacaaagattgcaa	sppR expression
sppRe3S	atgtcgacttaaaaaataatttcgacc	
sppR5	catacaaatacgagctcatacttgcgattact	sppR mutant complementation
sppR3	gtctttgccggtaccaaccccctttaaaaaataa	
dFNR-Xh5	ttctcgaggaggaaactgatagattag	deletion of Fnr-box except the "tt", dF2
dFNR-Xh3	ttctcgagaagaaatactccatcttttg	
mFNR3.53b	ttacagaaagttacaggaggaaactgatagattag	site-directed mutations of Fnr-box, dFm2
mFNR5.3c	tcgagtaactttctgtaagaaatactccatcttttg	
gtfB fw	agcaatgcagccatctacaaat	RealTime-qPCR
gtfB rv	acgaactttgccgttattgtca	RealTime-qPCR
gbpC fw	atacacaagcatcagaac	RealTime-qPCR
gbpC rv	attagcattagccttagc	RealTime-qPCR
brpA fw	cgtgaggtcatcagcaaggtc	RealTime-qPCR
brpA rv	cgctgtaccccaaaagtttagg	RealTime-qPCR
16S rRNA fw	cacaccgcccgtcacacc	RealTime-qPCR
16S rRNA rv	cagccgcaccttccgatacg	RealTime-qPCR
sppR-LR	gatcaatagcctctttcgaaatagttgtccttgc	frameshift *sppR* mutant, sppRf
sppR-RF	gcaaggacaactatttcgaaagaggctattgatc	frameshift *sppR* mutant, sppRf
sppR-RR	agaaggataaataccagaaatagtaagtaaacgag	frameshift *sppR* mutant, sppRf
sppR-LF	actaatccagtaccaaatctatcttgtcg	frameshift *sppR* mutant, sppRf
sppR-RR2	aggataaataccagaaatagtaagtaaacgag	Overalpping PCR for sppRf
sppR-LF2	accaaatctatcttgtcgttatttcacacattc	Overalpping PCR for sppRf
sppR-sc5	ttgcttgataggcaaggacaactatt	screening of sppRf
sppR-sc3	aagagaagtttgtgaataggaatagc	screening of sppRf
sppR-sq	acttggcagttctatttgttaagg	sequencing confirmation of sppRf
sppR-B1mF	aacttgatgtaatccttaggaggaaactgatag attag	mutation of SppR-box 1 via Q5 kit
sppR-B1mR	aaactccatatattgtttttgagccgttcattc	mutation of SppR-box 1 via Q5 kit
sppR-B2mF	aagtgatgttaatagcacatagcgaaatg atacg	mutation of SppR-box 2 via Q5 kit
sppR-B2mR	tctctatcattaggccgactagcttacaatc	mutation of SppR-box 2 via Q5 kit
sppR-B2dF	gatgatgagcacatagcgaaatg atacg	deletion of SppR-box 2 via Q5 kit
sppR-B2dR	atcacttggccgactagcttacaatc	deletion of SppR-box 2 via Q5 kit
sppR-B1dF	gatgtttaccttaggaggaaactgatag attag	deletion of SppR-box 1 via Q5 kit
sppR-B1dR	aaactccttgtttttgagccgttcattc	deletion of SppR-box 1 via Q5 kit

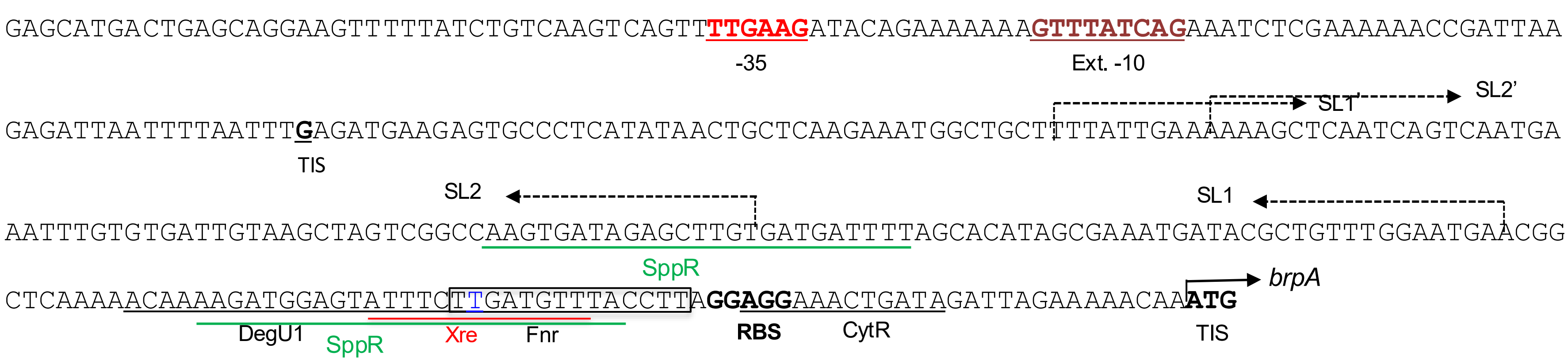

Figure S1. Relevant nucleotide sequences and features of the short *brpA* promoter. Underlined in dark brown and red faces are the predicted -10 and -35 regions. The transcription initiation site (TIS), the putative Shine-Dalgarno sequence (RBS), and translational initiation site ATG (TIS) are indicated in bold face. Potential binding sites for transcriptional regulators CytR, Fnr, Xre, SppR, and DegU are boxed or underlined, as indicated. Two inverted repeats, SL1-2, are illustrated by dashed arrows.

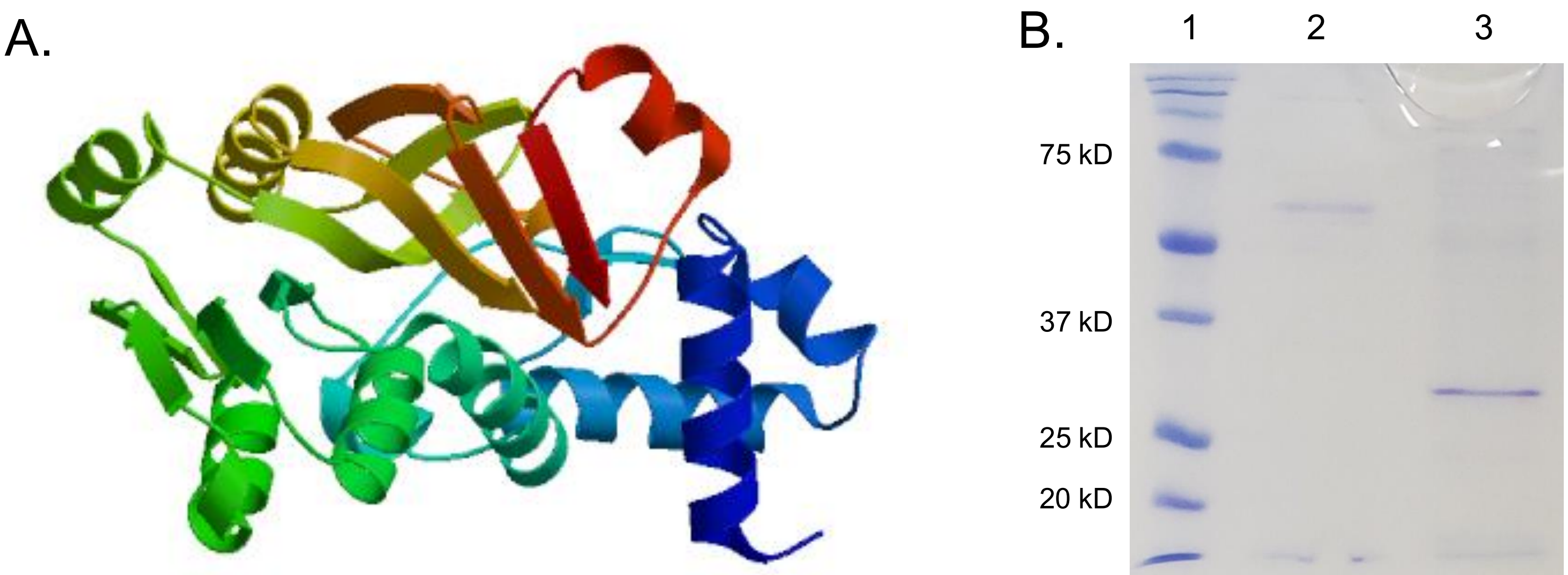

Figure S2. Protein domain prediction (**A**) and purification (**B**) of SppR. **A.**The SppR protein was predicted via AlphaFord v2 to possess a DeoR-type HTH-domain from aa1-58 (https://swissmodel.expasy.org/assess/7syXFU/01). **B.** rSppR was expressed in *E. coli* M15 as a his-tagged protein via pQE30 and purified under native conditions. Graph shows 15% SDS-PAGE analysis of the purified protein (lane 2, flow through, lane 3, elution fraction).

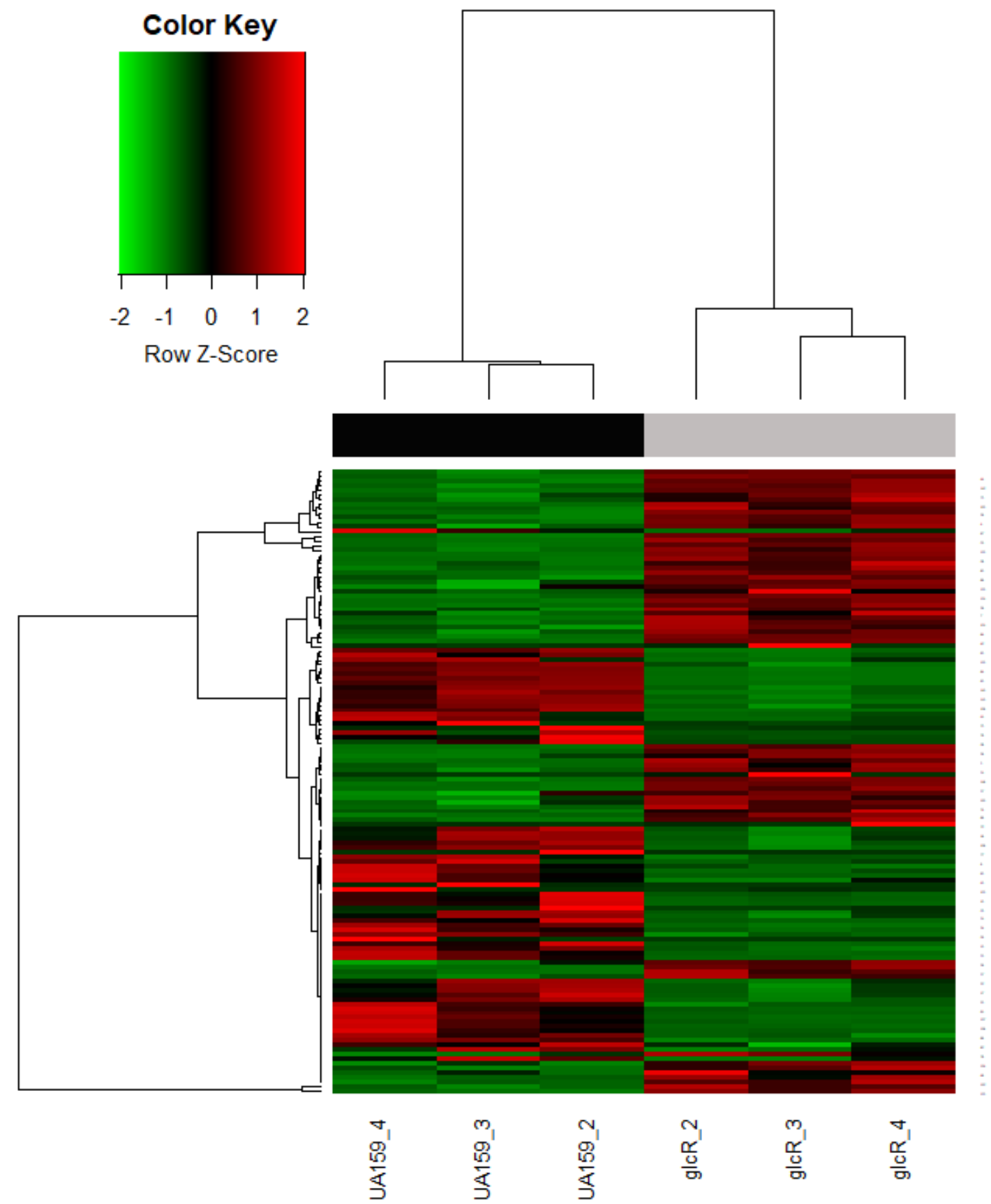

Figure S3. Differentially Expressed Genes Heatmap. It was created using the differentially expressed gene's normalized counts per million.
