# Supplementary material for: SppR in Streptococcus mutans coordinates stress tolerance response and BrpA expression
Source: Front Cell Infect Microbiol. 2026 Jul 27;16:1907905. doi: 10.3389/fcimb.2026.1907905 (PMC13454231; doi:10.3389/fcimb.2026.1907905)
Supplement: Supplementary file 2 [file SupplementaryFile2.pdf]

Table 2a. Down-regulated genes in response to *sppR* deficiency*

NCBI Locustag	Gene name/ Genbank tag	Description	Fold change (W/S)	P Value
SMU_RS00420	SMU_73	PFL family protein	2.912308191	0.024670011
SMU_RS00525	nigB	PTS system mannose/fructose/N-acetylgalactosamine-transporter subunit IIB	2.72686821	0.000190661
SMU_RS00530	nigC	PTS mannose/fructose/sorbose/N-acetylgalactosamine transporter subunit IIC	2.043888546	0.00348028
SMU_RS00700	mleS	malolactic enzyme	4.23334872	0.0000218
SMU_RS00705	mleP	AEC family transporter	4.659144438	0.000115112
SMU_RS00710	oxdC	oxalate decarboxylase family bicupin	4.736293516	0.000049
SMU_RS00715	gshR	dihydrolipoyl dehydrogenase family protein	4.17760084	0.0000868
SMU_RS00720	SMU_141	YoaK family protein	3.306865156	0.0000684
SMU_RS00885	SMU_179	NADPH-dependent FMN reductase	2.155070695	0.000250201
SMU_RS01020	SMU_194c	ASCH/PUA domain-containing protein	2.279905422	0.038852302
SMU_RS01025	SMU_195c	hypothetical protein	2.379539663	0.036603804
SMU_RS01030	SMU_196c	phage tail tip lysozyme	2.71045804	0.014087832
SMU_RS01035	SMU_197c	hypothetical protein	2.916460527	0.012879101
SMU_RS01040	tpn	ATP-binding protein	2.535828164	0.029861114
SMU_RS01045	SMU_199c	conjugal transfer protein	3.394894512	0.007100065
SMU_RS01055	SMU_201c	conjugal transfer protein	2.901983732	0.030353664
SMU_RS01065	SMU_205c	hypothetical protein	2.253199023	0.044609921
SMU_RS01075	SMU_207c	replication initiation factor domain-containing protein	2.530628803	0.019897448
SMU_RS01080	SMU.208c	FtsK/SpoIIIE domain-containing protein	2.684483624	0.005738901
SMU_RS01085	SMU_209c	DUF961 family protein	2.353168433	0.0179913
SMU_RS01090	SMU_210c	hypothetical protein	2.557876476	0.011608806
SMU_RS02390	comFA	DEAD/DEAH box helicase	10.62084841	0.000000227
SMU_RS02440	sppR	sugar-phosphate phosphatase transcriptional regulator SppR	4.397193115	0.00000743
SMU_RS02445	sppA	fructose-phosphate phosphohydrolase SppA	12.91001285	4.86E-09
SMU_RS02895	bsp	SH3 domain-containing protein	2.474516014	0.001825325
SMU_RS02950	comEA	helix-hairpin-helix domain-containing protein	7.15533629	0.000000205

SMU_RS02955	comEC	DNA internalization-related competence protein ComEC/Rec2	3.611469409	0.005341179
SMU_RS03145	argJ	bifunctional ornithine acetyltransferase/N-acetylglutamate synthase	2.766580423	0.026312243
SMU_RS03875	lytF	GBS Bsp-like repeat-containing protein	2.127169439	0.000271072
SMU_RS04060	tpn	IS3 family transposase	6.648952334	0.005873866
SMU_RS04115	galK	galactokinase	2.122605516	0.001799544
SMU_RS04510		prolyl oligopeptidase family serine peptidase	21.46139246	0.002355438
SMU_RS04605	dprA	DNA-processing protein DprA	8.504707544	0.0000014
SMU_RS05020	apbE	FAD:protein FMN transferase	2.103781679	0.002826516
SMU_RS05455	mtlA	PTS mannitol transporter subunit IICB	3.720231012	0.007608827
SMU_RS05825	hisF	imidazole glycerol phosphate synthase subunit HisF	5.363007054	0.018769869
SMU_RS05840	SMU_1267c	YrdB family protein	14.18804468	0.000792146
SMU_RS06125		IS982 family transposase	3.289920726	0.001710014
SMU_RS06130	mubP	mutanobactin A biosynthesis phosphopantetheinyl transferase MubP	9.722465235	0.000341718
SMU_RS06135	mubJ	mutanobactin A biosynthesis reductase MubJ	10.26938036	0.00021992
SMU_RS06140	mubI	mutanobactin A biosynthesis transacylase MubI	13.03238623	0.000115888
SMU_RS06145	mubM	mutanobactin A biosynthesis alpha/beta hydrolase MubM	17.97975258	0.0000627
SMU_RS06150	mubZ	mutanobactin A system MFS transporter MubZ	23.21530671	0.0000435
SMU_RS06245	SMU_1367c	class I SAM-dependent methyltransferase	21.52211054	0.000000791
SMU_RS06485	pdhD	dihydrolipoyl dehydrogenase	7.132071751	0.000978738
SMU_RS06490	clpB	ATP-dependent Clp protease ATP-binding subunit	4.843806982	0.000428727
SMU_RS06775	lacF	PTS lactose transporter subunit IIA	2.071306035	0.027101999
SMU_RS06780	lacD	tagatose-bisphosphate aldolase	12.42767542	0.002596757
SMU_RS06785	lacC	tagatose-6-phosphate kinase	2.201356724	0.001706715
SMU_RS06790	lacB	galactose-6-phosphate isomerase subunit LacB	2.054098684	0.002171205
SMU_RS06795	lacA	galactose-6-phosphate isomerase subunit LacA	2.169778431	0.004188146
SMU_RS07320	SMU_1613c	dephospho-CoA kinase	2.661726113	0.035093134
SMU_RS08020	cas2	CRISPR-associated endonuclease Cas2	2.015789199	0.000757315
SMU_RS08025	cas1c	type I-C CRISPR-associated endonuclease Cas1c	2.166525252	0.000178144
SMU_RS08030	cas4	CRISPR-associated protein Cas4	2.486729006	0.0000717

SMU_RS08035	cas7c	type I-C CRISPR-associated protein Cas7/Csd2	2.732012378	0.00000541
SMU_RS08040	cas8c	type I-C CRISPR-associated protein Cas8c/Csd1	2.538951005	0.00000803
SMU_RS08045	cas5c	type I-C CRISPR-associated protein Cas5c	2.484128598	0.00000838
SMU_RS08050		CRISPR-associated helicase/endonuclease Cas3	2.088850228	0.0000358
SMU_RS08500	hdrR	LytTR family transcriptional regulator HdrR	2.031219279	0.009620579
SMU_RS09005	SMU_1980c	competence type IV pilus minor pilin ComGG	8.491187147	0.00000233
SMU_RS09010	comGF	competence type IV pilus minor pilin ComGF	7.362973479	0.00000151
SMU_RS09015	SMU_1982c	competence type IV pilus minor pilin ComGE	7.778925883	0.00000224
SMU_RS09020	comYD	competence type IV pilus minor pilin ComGD	8.519307008	0.000000106
SMU_RS09025	comYC	competence type IV pilus major pilin ComGC	7.297182234	0.000000183
SMU_RS09030	comYB	competence type IV pilus assembly protein ComGB	9.761936918	0.000000122
SMU_RS09035	comYA	competence type IV pilus ATPase ComGA	4.676609663	0.00000531
SMU_RS09720	gabD	NAD-dependent succinate-semialdehyde dehydrogenase	2.10092866	0.002889275

*Results were expressed as transcript ratios of the wild-type (W) over the sppR mutant (S).

Table 2b. Up-regulated genes in response to *sppR* deficiency*

NCBI Locus tag	Gene name / GenBank ID	Description	Fold Change (S/W)	P Value
SMU_RS02835	pyrD	dihydroorotate oxidase	6.517676068	3.01E-09
SMU_RS03570		IS3-like element ISSmu1 family transposase	5.702970922	0.0174753
SMU_RS03000	SMU_635	VIT1/CCC1 transporter family protein	5.129250943	1.62E-08
SMU_RS05795	SMU_1258c	restriction endonuclease	4.772676238	0.014533
SMU_RS04500	licT	BglG family transcription antiterminator LicT	4.540486036	2E-08
SMU_RS00485	SMU_87	TIGR01440 family protein	4.532348025	6.89E-07
SMU_RS10070		hypothetical protein	4.212002361	0.0001898
SMU_RS10140	SMU_1395c	hypothetical protein	4.122481583	0.0000276
SMU_RS07370	SMU_1623c	CvfB family protein	3.882604574	5.84E-08
SMU_RS01455	SMU_293	HXXEE domain-containing protein	3.402689364	2.94E-06
SMU_RS02610	SMU_543	rhodanese-like domain-containing protein	3.392946901	3.01E-08
SMU_RS06360	gbpC	glucan-binding protein GbpC	3.390821157	0.0000779
SMU_RS10050		type II toxin-antitoxin system PemK/MazF family toxin	3.220339676	0.0050493
SMU_RS05420	atmA	amino acid ABC transporter substrate-binding protein	3.030976357	7.57E-07
SMU_RS07420	SMU_1634c	NUDIX hydrolase	2.99759508	5.33E-08
SMU_RS03930	SMU_848	ribosomal-processing cysteine protease Prp	2.953731454	2.43E-06
SMU_RS02620	SMU_547	DUF3165 family protein	2.944795104	2.84E-07
SMU_RS03935	rpmA	50S ribosomal protein L27	2.930797912	4.89E-06
SMU_RS04620	gtfB	glucosyltransferase GtfB	2.831675777	0.000055
SMU_RS00875	SMU_177	PTS sugar transporter subunit IIC	2.828492395	0.0218822
SMU_RS06075	SMU_1319c	esterase family protein	2.804115998	0.0001486
SMU_RS04595	fatB	siderophore ABC transporter substrate-binding protein	2.764199141	1.89E-07
SMU_RS02200	SMU_457	hypothetical protein	2.728317705	1.04E-06
SMU_RS06905	glnP	amino acid ABC transporter permease	2.652341441	6.22E-06
SMU_RS09155	secY	preprotein translocase subunit SecY	2.63392642	3.54E-06
SMU_RS05875	ezrA	septation ring formation regulator EzrA	2.616165902	0.0000426

SMU_RS05755	SMU_1249c	DUF4352 domain-containing protein	2.603161555	0.0001311
SMU_RS05740	SMU_1245c	YjjG family noncanonical pyrimidine nucleotidase	2.582708789	4.12E-06
SMU_RS08275	SMU_1803c	DUF4230 domain-containing protein	2.563435893	0.0001932
SMU_RS06410	#N/A	IS3-like element ISSmu1 family transposase	2.55652237	0.0056069
SMU_RS08845	SMU_1946	DUF4956 domain-containing protein	2.519493878	2.15E-07
SMU_RS04705	pycB	oxaloacetate decarboxylase subunit alpha	2.507001925	3.4E-07
SMU_RS02355	pflC	glycyl-radical enzyme activating protein	2.486639553	7.46E-06
SMU_RS04735		cysteine-rich KTR domain-containing protein	2.477403516	0.0000215
SMU_RS04730	SMU_1028	alpha/beta fold hydrolase	2.47581193	0.0000216
SMU_RS08215	SMU_1789c	YebC/PmpR family DNA-binding transcriptional regulator	2.46446479	0.0000168
SMU_RS04495	potD	ABC transporter substrate-binding protein	2.401904422	0.0000225
SMU_RS02550	pheA	chorismate mutase	2.37274018	0.0014528
SMU_RS04960	SMU_1076	ECF transporter S component	2.371082935	0.0000421
SMU_RS00435	ampD	glycoside hydrolase family 73 protein	2.369380372	0.0000291
SMU_RS02975	SMU_630	serine hydrolase	2.325012676	5.39E-06
SMU_RS03765	mutT	8-oxo-dGTP diphosphatase	2.314003655	0.0153988
SMU_RS02545	SMU_530c	DUF975 family protein	2.309386468	0.000649
SMU_RS02195	mraY	phospho-N-acetylmuramoyl-pentapeptide- transferase	2.301705218	0.0001252
SMU_RS07015	SMU_1546	GtrA family protein	2.223312441	0.0000139
SMU_RS06620	rmlB	dTDP-glucose 4,6-dehydratase	2.205399858	1.85E-06
SMU_RS00510	pyrG	CTP synthase	2.181863474	0.0000467
SMU_RS08540	mutY	A/G-specific adenine glycosylase	2.181174881	0.0000141
SMU_RS06055	SMU_1315c	ABC transporter ATP-binding protein	2.179295448	0.0004074
SMU_RS05780	SMU_1254	HAD family hydrolase	2.170256666	0.0000612
SMU_RS04215	SMU_911c	DUF308 domain-containing protein	2.155658255	0.0012083
SMU_RS03350	SMU_711	DUF4430 domain-containing protein	2.152657618	0.0008752
SMU_RS06290	leuD	3-isopropylmalate dehydratase small subunit	2.141328137	0.0046904
SMU_RS08980	SMU_1975c	type II CAAX endopeptidase family protein	2.13233796	0.000069
SMU_RS01415	SMU_283	Blp family class II bacteriocin	2.128827916	0.0002364

SMU_RS05800	SMU_1259	Eco57I restriction-modification methylase domain-containing protein	2.126193776	0.0107887
SMU_RS02585	trpA	tryptophan synthase subunit alpha	2.122924811	0.0001329
SMU_RS00585	tagR	MurR/RpiR family transcriptional regulator	2.113062489	0.0016532
SMU_RS09040	SMU_1988c	DUF1033 family protein	2.095954666	0.0017219
SMU_RS07045	SMU_1553c	hypothetical protein	2.084360684	0.0034741
SMU_RS04365	pnuC	nicotinamide riboside transporter PnuC	2.082770473	0.0003093
SMU_RS05760	SMU_1250c	hypothetical protein	2.081227825	0.023879
SMU_RS08250	SMU_1798c	bis(5'-nucleosyl)-tetraphosphatase (symmetrical) YqeK	2.050281585	0.0000838
SMU_RS08855	secE	preprotein translocase subunit SecE	2.049440829	0.0045892
SMU_RS07655	Orf11	DUF1803 domain-containing protein	2.04226229	0.0025551
SMU_RS07395	SMU_1628	DUF3397 domain-containing protein	2.040152232	0.0004909
SMU_RS05135	SMU_1116c	hypothetical protein	2.029621366	0.0003213
SMU_RS08645	SMU_1895c	Blp family class II bacteriocin	2.003412902	0.0002764

* Results were expressed as transcript ratios of the sppR mutant (S) over the wild-type (W).
